# Initial success in the identification and management of the coronavirus disease 2019 (COVID-19) indicates human-to-human transmission in Wuhan, China

**DOI:** 10.7150/ijbs.45018

**Published:** 2020-04-06

**Authors:** Annoor Awadasseid, Yanling Wu, Yoshimasa Tanaka, Wen Zhang

**Affiliations:** 1 Lab of Chemical Biology and Molecular Drug Design, College of Pharmaceutical Science, Zhejiang University of Technology, Hangzhou, 310014, China; 2 Lab of Molecular Immunology, Virus Inspection Department, Zhejiang Provincial Center for Disease Control and Prevention, Hangzhou, 310051, China; 3 Center for Natural Products Research, Chengdu Institute of Biology, Chinese Academy of Sciences, Chengdu 610041, PR China.; 4 Center for Medical Innovation, Nagasaki University, 1-7-1 Sakamoto, Nagasaki 852-8588, Japan.

**Keywords:** COVID-19, SARS-CoV-2, infection, treatment, prevention

## Abstract

Coronavirus (CoV) has been one of the major pandemic threats to human health in the last two decades. The human coronavirus was first identified in 1960s. CoVs 229E, NL63, OC43, HKU1, SARS-CoV, and MERS-CoV have caused numerous disasters or human deaths worldwide. Recently, an outbreak of the previously unknown deadly CoV disease 2019 (COVID-19) caused by Severe Acute Respiratory Syndrome CoV 2 (SARS-CoV-2, early named 2019-nCoV) occurred in Wuhan, China, and it had caused 81238 cases of confirmed infection, including 3250 deaths until March 19, 2020. Its risks and pandemic potential have brought global consideration. We summarized epidemiology, virological characteristics, clinical symptoms, diagnostic methods, clinical treatments, and prevention methods for COVID-19 to present a reference for the future wave of probable CoV outbreaks.

## Background

On December 31, 2019, the Health Commission of Hubei Province, China, first declared a cluster of unexplainable cases of pneumonia 1. Subsequently, on January 10, 2020, the World Health Organization (WHO) announced that there was an outbreak of pneumonia of undiscovered cause in the city of Wuhan, Hubei Province, China. With an epidemiological association to the Huanan Seafood Wholesale Market where birds, bats, snakes, and other wildlife animals are sold 2. A previously unknown coronavirus (CoV) was immediately discovered in the specimens from the patients. Which WHO provisionally named as the 2019 novel coronavirus (2019-nCoV). Later on February 11, 2020, WHO officially announced that the novel coronavirus was renamed as Severe Acute Respiratory Syndrome CoV 2 (SARS-CoV-2) and named the disease caused by SARS-CoV-2 as COVID-19 that stands for the CoV disease 2019.

**Figure 1:**
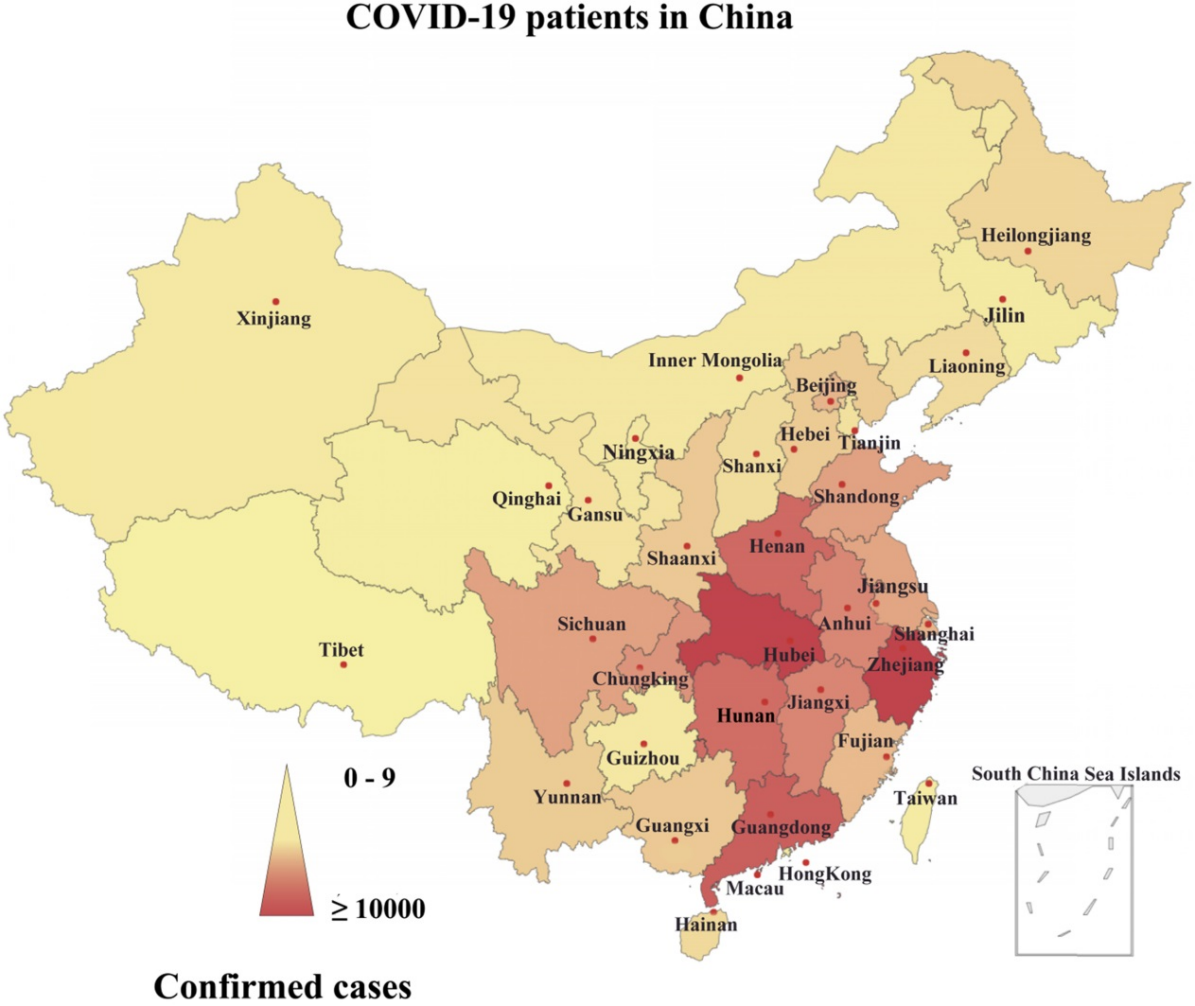
** Geographical distribution of the confirmed cases of COVID-19 in China.** Note: the statistical data as of March 19, 2020.

Since December 31, 2019 and as of March 19, 2020-6.00 PM GMT, overall, 81238 laboratory-confirmed cases of COVID-19, and 3250 deaths were announced in all over China, especially in Wuhan, Hubei Province (Fig. 1) 3. Most of the fatal cases had typical symptoms such as fever, dry cough, dyspnoea, and radiological findings of bilateral lung infiltrates several days after the start of the initial symptoms 4. Subsequent etiological studies showed that the patients were infected with SARS-CoV-2 4. This is the first report that SARS-CoV-2 can infect humans. In addition, SARS-CoV-2 displays strong ability of human-to-human transmission worldwide; although it currently has low pathogenicity, COVID-19 may lead to human death” 1. As a result, it raises global public health concerns about its risks and pandemic potential.

## Epidemiology

In December 2019, Wuhan, in China's Hubei Province, became the epicenter of an unexplained outbreak of pneumonia, drawing widespread attention not only in China but also in other countries 5. As of March 19, 2020, 81238 laboratory-confirmed human cases in China, 131320 confirmed cases in 153 countries, and other 696 confirmed cases on an international conveyance in Japan have been reported to WHO (Table 1) 3. Confirmed cases of Wuhan passengers were announced in Thailand on January 13, in Japan on January 15 and in South Korea on January 19, and so on 6. It was soon found that SARS-CoV-2 caused COVID-19 and was epidemiologically related to the Huanan Seafood market in Wuhan 7. Where restaurants supply wild animals, suggesting that the main route of SARS-CoV-2 transmission may be the point-source zoonotic (animal-to-human) route 8. According to the "diagnosis and treatment of pneumonia" program of COVID-19, the main source of infection after the initial transmission to patients was SARS-CoV-2 infection in patients 8. Recent studies suggest that SARS-CoV-2 may be transmitted from person to person primarily through respiratory droplets and through contact 9-11. Compared to SARS-CoV and MERS-CoV, SARS-CoV-2 in Wuhan currently appears to be less pathogenic and safe if we assumed that the virus spreads effectively worldwide 12. Still, the possibility of SARS-CoV-2 mutation cannot be ruled out 13. The incubation period of SARS-CoV-2 is about 7 days on average, the short one is 2-3 days, and the long one could be over 21 days 4, 14. Referring to the incubation period for other CoVs, the SARS-CoV-2 case was placed under close contact medical observation for 14 days, and close contacts were placed under medical observation in the hospital. After 14 days, if there is no evidence of the disease, the person can be determined as not infected 4, 14. Everybody is susceptible to SARS-CoV-2, and disease in immunocompromised individuals progress relatively faster and more severe 2, 14. Children have less exposure and a lower risk of infection; in the same way, the elderly, patients with chronic diseases, and people with weak immunity are more likely to be infected 4, 14.

SARS-CoV-2 belongs to the new coronavirus species in the genus containing SARS-CoV and MERS-CoV 15. It is enveloped, and the particles are round or elliptic, usually pleomorphic, with a diameter of 60~140 nm 16, 17. Its genetic characteristics are different from those of SARS-CoV and MERS-CoV 18. Current studies have shown that SARS-like coronavirus in bat (bat-SL-CoVZC45, MG772933.1) has over 85% homology to SARS-CoV-2 16. SARS-CoV-2 can be found in human respiratory epithelial cells at around 96 hours after infection 14, 16. In contrast, it takes about 6 days to isolate the viruses when Vero E6 and Huh-7 cell lines are used in the *in-vitro* culture system 14, 16. CoVs are single-stranded RNA viruses that mutate readily, making it challenging to develop sustained immunity to them 19. Flu viruses, for example, circulate every year and require the latest vaccine because the virus type often changes 20.

## Virology

### Phylogenetic analysis

**Figure 2:**
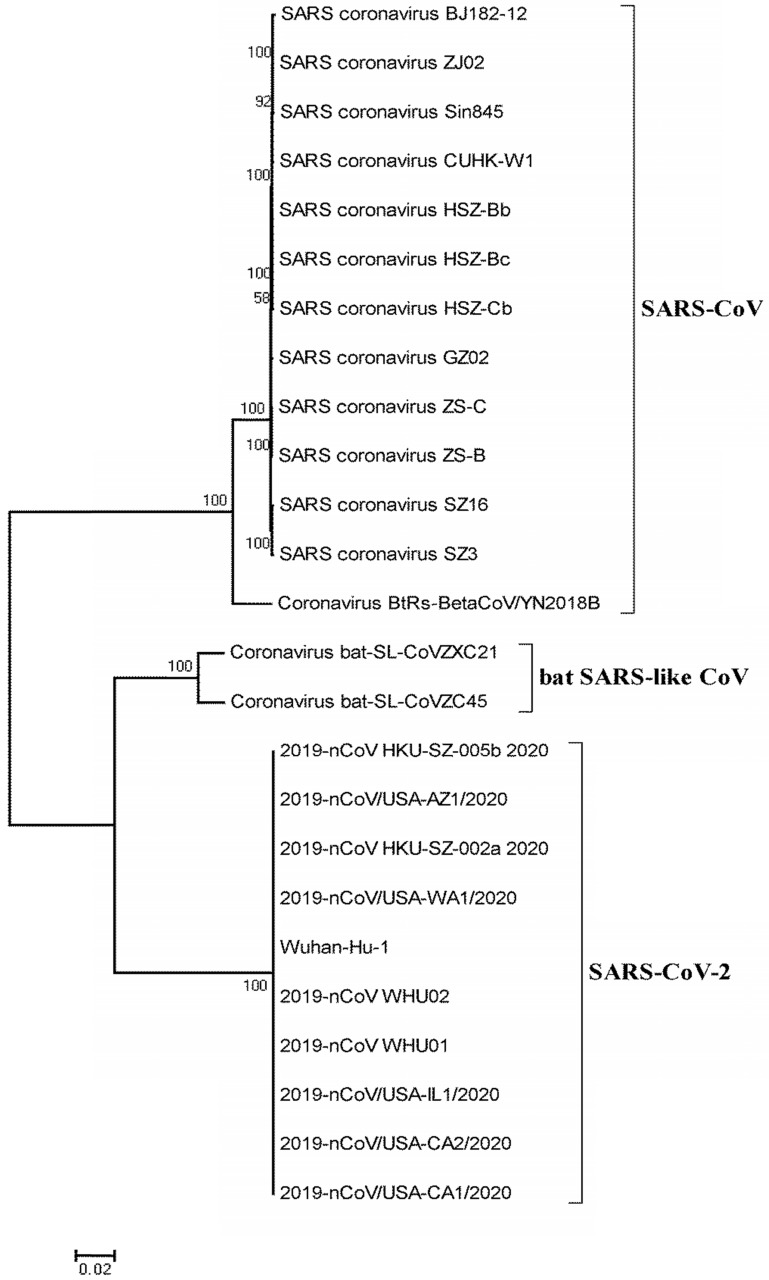
**Phylogenetic relationship of SARS-CoV-2 with bat SARS-like CoV and SARS-CoV.** The full genome sequences of SARS-CoV-2 and SARS-CoV were obtained from the National Center for Biotechnology Information search engine (http://www.ncbi.nim.nih.gov/). The phylogenetic tree was created using the MEGA 5.0 software package and neighbor-joining program (http://www.megasoftware.net) with 1000 boots trapped value support and a Poisson correction. The bootstrap values greater than 50% are given at the nodes. The scale bar shows the phylogenetic range determined from the number of variations. The NCBI GenBank accession numbers of the genome sequences are EU371564.1 (SARS coronavirus BJ182-12), EU371559.1 (SARS coronavirus ZJ02), AY559093.1 (SARS coronavirus Sin845), AY278554.2 (SARS coronavirus CUHK-W1), AY394985.1 (SARS coronavirus HSZ-Bb), AY394994.1 (SARS coronavirus HSZ-Bc), AY394986.1 (SARS coronavirus HSZ-Cb), AY390556.1 (SARS coronavirus GZ02), AY395003.1 (SARS coronavirus ZS-C), AY394996.1 (SARS coronavirus ZS-B), AY304488.1 (SARS coronavirus SZ16), AY304486.1 (SARS coronavirus SZ3), MK211376.1 (Coronavirus BtRs-BetaCoV/YN2018B), MG772933.1 (Coronavirus bat-SL-CoVZC45), MG772934.1 (Coronavirus bat-SL-CoVZXC21), MN975262.1 (2019-nCoV HKU-SZ-005b 2020), MN997409.1 (2019-nCoV/USA-AZ1/2020), MN938384.1 (2019-nCoV HKU-SZ-002a 2020), MN985325.1 (2019-nCoV/USA-WA1/2020), NC_045512.2 (Wuhan-Hu-1), MN988669.1 (2019-nCoV WHU02), MN988668.1 (2019-nCoV WHU01), MN988713.1 (2019-nCoV/USA-IL1/2020), MN994468.1 (2019-nCoV/USA-CA2/2020), and MN994467.1 (2019-nCoV/USA-CA1/2020).

The circulating CoV is a newly discovered CoV, named SARS-CoV-2 by WHO. People are susceptible to the virus because they lack immunity to new strains of CoV 2, 14. SARS-CoV-2 is different from any of the six known CoVs that can infect humans, including HCoV-229E, HCoV-OC43, HCoV-HKU1, HCoV-NL63, SARS-CoV, and MERS-CoV. It is the seventh CoV that can infect humans and cause severe pneumonia. Genomic analysis of SARS-CoV-2 showed that it was markedly different from both SARS-CoV and MERS-CoV, with the genomic difference being 21% and 48.2%, respectively 21. Despite being a β-CoV, SARS-CoV-2 is not the same as the SARS-CoV and MERS-CoV, and is considered to be a distant relative of SARS-CoV, rather than a variant of SARS-CoV or a resurgence of SARS-CoV 21. Previous studies showed that two complete virus genomes (HKU-SZ-002a and HKU-SZ-005b) were sequenced using Nanopore technology and revealed a SARS-CoV-2 is most closely related to bat SARS-like CoVs, bat-SL-CoVZXC21 (NCBI registration number MG772934) and bat-SL-CoVZC45 (NCBI registration number MG772933) 1, which belong to the *Betacoronavirus* genus (Fig. 2). The virus genome sizes of HKU-SZ-002a and HKU-SZ-005b were about 29.8 kbp, and the GC contents were 38%. The difference between HKU-SZ-002a and HKU-SZ-005b was only two bases 1. One of them is a non-synonymous mutation at the amino acid position of the unstructured protein at 336 (Ser336 for HKU-SZ-002a; Leu336 for HKU-SZ-005b) 1. Approximately 66% of the amino acid residues of the N-terminal domain of Spike subunit 1 of SARS-CoV-2 are identical to that of the SARS-related CoV. The core region of the SARS-CoV-2 receptor-binding domain is approximately 68% similar to the virus at the amino acid level. It has the same acidity as that of the SARS-related CoV. The amino acid sequences in the outer subdomain of the Spike subunit 1 receptor binding domain show only 39% identity, which may influence the selection of human receptors and thus the biological behavior of the virus 1.

### Molecular signatures of SARS-CoV-2

According to its genetic characteristics, the CoV family consists of four genera, including *Alphacoronavirus*, *Betacoronavirus*, *Gammacoronavirus*, and *Deltacoronavirus* 22. Of all RNA viruses, the CoV RNA genome (ranging from 26 to 32 kbp) is the largest one 23. SARS-CoV and MERS-CoV are classified into the *Betacoronavirus* genus and are zoonotic pathogens that can cause critical respiratory infections in humans 24. The SARS-CoV-2 genomic structure belongs to that of β-CoV (MN908947) 25. In the previous study in early 2020, samples were collected from seven patients with severe pneumonia who had been exposed to SARS-CoV-2 at a seafood market in Wuhan and were placed in an intensive care unit at the onset of the outbreak. The samples were then sent to the WIV laboratory for pathogen diagnosis 26. They observed five positive bands on PCR. A sample from bronchoalveolar lavage fluid (BALF) (WIV04) was analyzed using the next generation sequenced (NGS) to identify possible pathogens. Of the 1582 total readings obtained after filtering through 64 human genomes, 1,378 (87.1%) matched SARS-CoV sequences 26. The SARS-CoV-2 was named because the novel coronavirus has a 79.5% sequence identity with SARS-CoV BJ01 (GenBank accession number AY278488.2) 26. The full-length genome sequences of four SARS-CoV-2 (WIV02, WIV05, WIV06, and WIV07) (GISAID accession numbers EPI_ISL_402127-402130) were subsequently obtained from four other patients, with more than 99.9% homology being observed among them 26. The viral genome consists of six main open reading frames (ORFs) as well as many other accessory genes 26. Additional analysis showed that the SARS-CoV-2 genome had less than 80% nucleotide sequence identity with the SARS-CoV sequence. They obtained a short RdRp region from a bat CoV, known as RaTG13, that was previously found in *Rhinolophus affinis* in Yunnan Province, China, with a high degree of sequence identity with SARS-CoV-2 26. Physicochemical parameters of SARS-CoV-2 (Sequence ID: MN908947.3), including isoelectric point, instability index, estimated half-life, and grand average of hydropathicity (GRAVY), were investigated using the ProtParam tool of ExPASy 27. Analysis of physicochemical parameters showed that the SARS-CoV-2 polypeptide is 9194 amino acids with a molecular weight of 1069577.59 Dalton, theoretical pI: 9.02, the estimated half-life is 20 hours (mammalian reticulocytes, *in vitro*), 30 min (yeast, *in vivo*), and >10 hours (*Escherichia coli*, *in vivo*), and a GRAVY score of -0.087 GRAVY, categorizing the protein as unstable (Table 2).

Angiotensin-converting enzyme 2 (ACE2) is a membrane-bound aminopeptidase that has been shown to play a critical role in the cardiovascular and immune systems through two different important physiological mechanisms. ACE2 catalyzes the production of vasodilatory peptides, including angiotensin 1 to 7 28. Since its discovery in 2000, ACE2 has been extensively studied in the cardiovascular and metabolic fields, suggesting that it plays a critical role in the development of cardiac function, hypertension, and diabetes. In addition, it is noteworthy that ACE2 is a functional receptor for coronaviruses, such as SARS-CoV, and SARS infection is caused by the binding of the SARS-CoV protein trimer to the hydrophobic pockets of ACE2's extracellular catalytic domain 29. This interaction enables endocytosis, membrane fusion, and SARS-CoV entry into host cells. When the virus entered, ACE2 protein was downregulated, leading to a localized increase in Angiotensin II levels, which provided a molecular explanation for the continual development of Acute Respiratory Distress Syndrome (ARDS) during SARS-CoV infection. ACE2 is highly expressed in both heart and lung tissues. Therefore, in addition to lowering blood pressure and improving heart function, ACE2 inhibitors are expected to prevent ACE2/SARS-CoV protein interaction and inhibit SARS-CoV infection 29. The spike (S) protein of SARS-CoV-2 contains a SARS-CoV-like receptor binding domain, suggesting that its receptor should be ACE2, which is the same as one for SARS-CoV. Recently, it has been found that the expression level of ACE2 in Asian people is significantly higher than that in European and American people and that in men is higher than that in women 30-32. This difference also explains the high incidence of new coronary pneumonia in Asia, where there are more men than women 30-32. The SARA-CoV-2 primarily invades alveolar epithelial cells and interacts with ACE2, leading to respiratory symptoms in patients 33. When patients complicated with underlying cardiovascular diseases, the symptoms tended to be severe, which may be related to increased renin secretion in these patients and increased ACE2 secretion through negative feedback regulation, providing more binding sites for SARA-CoV-2 34.

### Clinical characteristics of SARS-CoV-2

**Figure 3:**
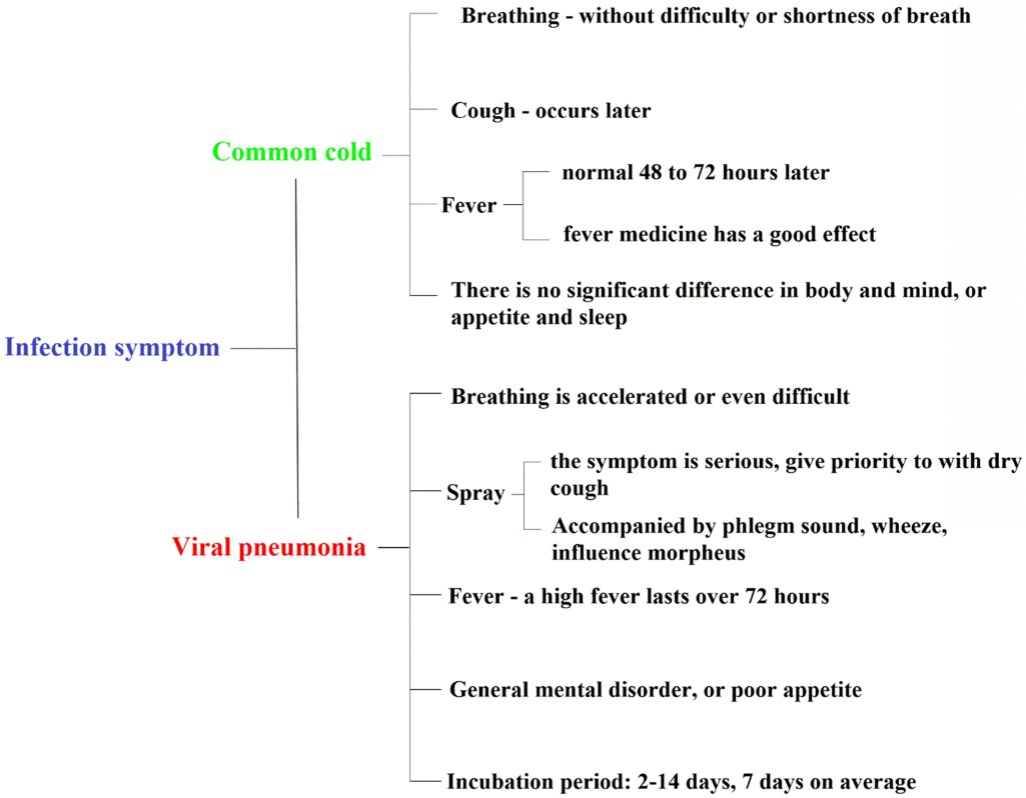
The difference between COVID-19 (SARS-CoV-2) and flu, common cold symptoms.

The common symptoms of COVID-19 are fever (91.7%), fatigue (75.0%), cough (75.0%), progressive dyspnea (36.7%), gastrointestinal symptoms (39.6%), and some patients (≤ 10%) start with mild symptoms or even no palpable fever (Fig. 3) 35. From the current case situation, most patients have a good prognosis; a few patients are in critical conditions or even died 36. In addition to the above symptoms, there may be "atypical" symptoms, such as (a) only the symptoms of the digestive system as the first manifestation: such as mild tolerance, weakness, general mental disorder, nausea and vomiting, diarrhea, etc.; (b) neurological symptoms as the first manifestation: such as headache; (c) cardiovascular symptoms as the first manifestation: such as palpitation, chest tightness, etc.; (d) ophthalmic symptoms as the first manifestation: such as conjunctivitis; (e) only mild muscle soreness in limbs or lower back 4.

Many respiratory diseases are characterized by fever, fatigue, dry cough, and other symptoms. Diagnosis of COVID-19 requires a physician to make a comprehensive judgment based on whether he/she has been to an endemic area before the onset of the disease, whether he/she has been exposed to suspected or confirmed cases, and the results of laboratory tests 14. A dry cough is one of the symptoms of COVID-19; the main difference between a dry cough and a cough is the presence of sputum 4, 14. A dry cough is a cough in which there is no sputum or very little sputum. Common cold, acute bronchitis, inhalation of some irritant gas, and dust and so on can also cause a dry cough. Pneumonia may be accompanied, but early pneumonia may not be fever, only chills and respiratory infection symptoms, but computed tomography (CT) scan will show pneumonia. In severe cases, dyspnea and hypoxemia usually occur 1 week after infection 4, 37. In severe cases, it rapidly progressed to ARDS, septic shock, metabolic acidosis that was difficult to treat, and bleeding and coagulation dysfunction 4, 36, 37. It is worth noting that patients with severe or critical illness may have a moderate or low fever or even no palpable fever 4. Patients with mild cases showed only low fever, mild fatigue, and no pneumonia usually recovered after 1 week 4. A few infected patients exhibited no obvious clinical symptoms and only tested positive 4. From the current case situation, most of the patients have a good prognosis, the child cases are relatively mild, and a few patients are in critical condition 14. Deaths are more common in the elderly and those with chronic underlying diseases 14. Of the 1023 deaths (fatality rate 2.3%), most were aged ≥ 60 years or had comorbidities such as hypertension, cardiovascular disease, and diabetes 38. Laboratory examination showed that the total number of white blood cells in peripheral blood was normal or decreased, the lymphatic size was reduced, and the cell count decreased. Liver enzymes, muscle enzymes, and myoglobin increased in some patients. Most patient's C-reactive protein (CRP) and erythrocyte sedimentation rate were elevated, and procalcitonin (PCT) was normal. Normal or slightly higher levels of cytokines, inflammatory cytokines such as interleukins-2 (IL-2), tumor necrosis factor-α (TNF- α), IL-6, interferon-γ (IFN-γ), and so on, can be markedly increased in patients with organ failure. In severe cases, *D-D* dimer increased, and lymphocytes progressively decreased 4, 36, 37.

## Diagnosis

On January 28, 2020, the fourth edition of the “Pneumonia Diagnostic Guidelines” for COVID-19 was officially released, and the pneumonia caused by SARS-CoV-2 was characterized by human-to-human transmission 39. According to the "Pneumonia Diagnosis and Treatment” program for COVID-19 (trial version 4)", suspected cases can be defined as (1) epidemiological history: travel history or residence history in Wuhan within two weeks before the onset of the disease; or had been in contact with patients with fever and respiratory symptoms from Wuhan within 14 days before the onset, or had aggregated onset. (2) clinical manifestations: fever; (a) with typical pneumonia imaging characteristics; (b) the total number of early-onset white blood cells normal or decreased, or lymphocyte count decreased. Real-time fluorescence quantitative PCR (RT-PCR) tested confirmed cases: based on meeting the criteria for suspected cases, sputum, pharynx swabs, lower respiratory tract secretions, and other specimens for positive SARS-CoV-2 nucleic acid, or viral gene sequencing, highly homologous to SARS-CoV-2 39. As one of the methods for early diagnosis of suspected cases, chest imaging is widely used in a clinic because of its convenience and versatility 14. It is easy to diagnose multiple ground glass density shadows in typical bilateral lungs 14, 40. However, COVID-19 caused by SARS-CoA-2 has not been thoroughly analyzed, clinical features have not been described collectively 14, 41.

**Figure 4:**
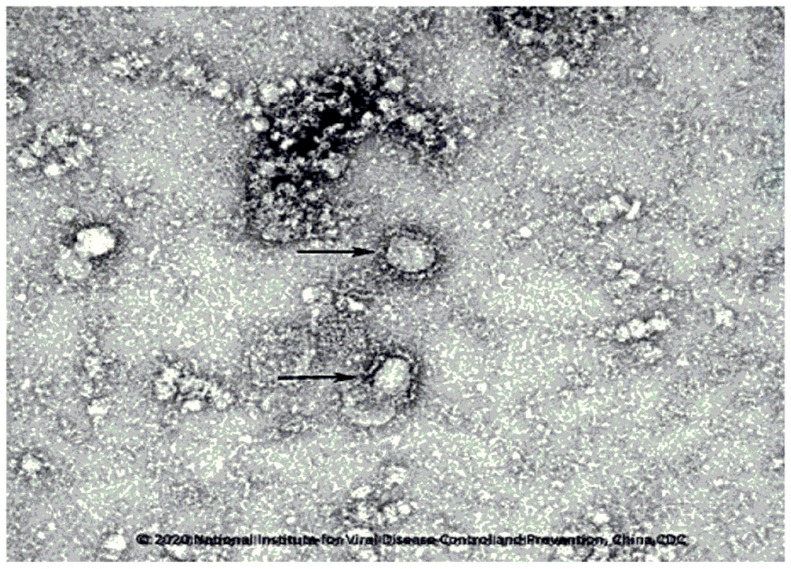
SARS-CoV-2 particles in ultrathin sections of human airway epithelial cells.

In the early stage of the disease, it was mainly manifested as scattered small flakes in the lung, and focal ground-glass density and consolidation shadows distributed under the pleura or along the bronchial tree 14. In the progressive stage, most of the patients reached the scene of the most severe lung infiltration within 8-14 days after the onset, and the ground glass density shadows could be fused into scattered and multiple consolidation shadows, in which air bronchi signs could be seen 4. When compared to SARS in 2003, the main manifestations of COVID-19 are: fever is evident at the initial stage (2-3 days after onset), and the imaging manifestations are usually small lamina in the lung, a ground-glass shadow is common 4, 14. The distribution of the following lung and surrounding areas is universal 4, 14. Progressive exacerbation occurs within 5-8 days after the onset of the disease, and the most severe symptoms appear 2-3 weeks after onset, which is called a progressive stage 42. Ground glass shadow consolidation and mesh shadow appear, followed by interlobular septum thickening 42. Small patchy lesions develop to large patchy ones, with single progression to multiple or diffuse lesions. The development is swift, and the change is rapid 14. The number of affected pulmonary lobes is increased rapidly, and pathological changes can progress from a lung to several lobes, from one lung to two lungs, even "white lung" appears 14. Two to three weeks after the onset of the disease, it enters the absorption phase and changes from multiple and diffuse lesions to the limitation 14. The density begins to decrease, and the range gradually shrinks or even disappears. After inflammatory absorption, some patients leave behind some manifestations of pulmonary interstitial fibrosis 4, 14. This new type of viral pneumonia is fierce, and the characteristics of human-to-human transmission have been identified (China's National Microbiology Data Center shared the image 43, 44, Fig. 4). "Early detection, early report, early isolation, and early treatment" is critical 45. The recognition of atypical imaging manifestations plays a vital role in the early identification of COVID-19 caused by SARS-CoV-2 45. It is recommended to timely conduct multiple examinations and follow up with imaging to quickly and appropriately perform the diagnosis and observe the changes in the disease 45.

## Treatment and Prevention

### Treatment recommendations

Principles of treatment for suspected and confirmed cases should be isolated in designated hospitals with sufficient isolation conditions and protective conditions 14. Suspected cases should be treated in isolation, while confirmed cases can be admitted to the same ward. Critical cases should be admitted to the ICU as soon as possible 14. Bed rest, vital signs (heart rate, pulse oxygen saturation, respiratory rate, and blood pressure) should be observed. Supportive treatment should be strengthened to ensure adequate heat to maintain a stable internal environment such as water, electrolyte, and acid-base balance 14. Complete blood count, C-reactive protein (CRP), procalcitonin (PCT) and dirt were monitored according to the conditions of organ functions (liver enzyme, bilirubin, myocardial enzyme, creatinine, urea nitrogen, urine volume, etc.), coagulation function, arterial blood gas analysis, and chest imaging 4. Effective oxygen therapy, including a nasal catheter, mask oxygen, high flow oxygen therapy (HFNO), and non-invasive mechanical ventilation (NIV) or invasive mechanical ventilation is recommended 14. Extracorporeal oxygenation (ECMO) should be considered for refractory hypoxemia that is difficult to manage after protective pulmonary ventilation 4.

### Therapeutic use

There is no evidence from a randomized controlled trial (RCT) to support specific anti-SARS-CoV-2 drugs for suspected or confirmed cases up to now. In treating Severe Acute Respiratory Syndrome (SARS) and the Middle East respiratory syndrome (MERS) infections, some studies, such as retrospective cohort studies, historical control studies, case reports, or serial case reports, confirmed that lopinavir/ritonavir or, in combination with antivirals, has some therapeutic advantages, such as reducing the incidence of acute respiratory distress syndrome (ARDS) mortality 46-48. Previous published systematic reviews show that lopinavir/ritonavir is primarily effective in early application, and can reduce mortality and glucocorticoid dosage 49, 50. If the early treatment window is missed, however, the late application has no significant effect. We still need to carry out real-world clinical studies to further explore the clinical implications of their early use in the COVID-19 14. The effectiveness of antiviral combinations remains controversial 51-54. Therefore, blind or inappropriate use of antibiotics, especially in combination with broad-spectrum antibiotics, should be avoided in the treatment of COVID-19. Strengthening bacteriological monitoring, when there are secondary bacterial infections appropriate, antimicrobial agents may timely be applied. Depending on the patient's clinical presentation, patients with mild cases can take oral antibiotics and antimicrobial agents, such as amoxicillin, azithromycin, or fluoroquinolones, against community-acquired pneumonia if the bacterial infection cannot be ruled out. Severe patients need to cover all possible pathogens for empirical treatment, after the pathogen identified descending stair therapy 51, 52. Steroid therapy for severe ARDS is controversial, thus, systemic use of glucocorticoids should be cautious. Methylprednisolone can be used as appropriate in patients with rapid development or severe illness, and 40~80 mg daily can be considered depending on the severity of the disease, with the total daily dose not exceeding 2 mg/kg 55-57. Previous studies on SARS showed that timely noninvasive continuous positive airway pressure ventilation and corticosteroids were used when lung shadows increase and dyspnea worsens effective solutions. Appropriate use of glucocorticoids can significantly improve the clinical symptoms of SARS patients, reduce the extent of disease progression, accelerate pulmonary disease, and absorption, but failed to shorten the length of hospitalization 55, 56. Steroid therapy has a specific incidence of adverse reactions 57.

### Possible new therapies for COVID-19

**Figure 5:**
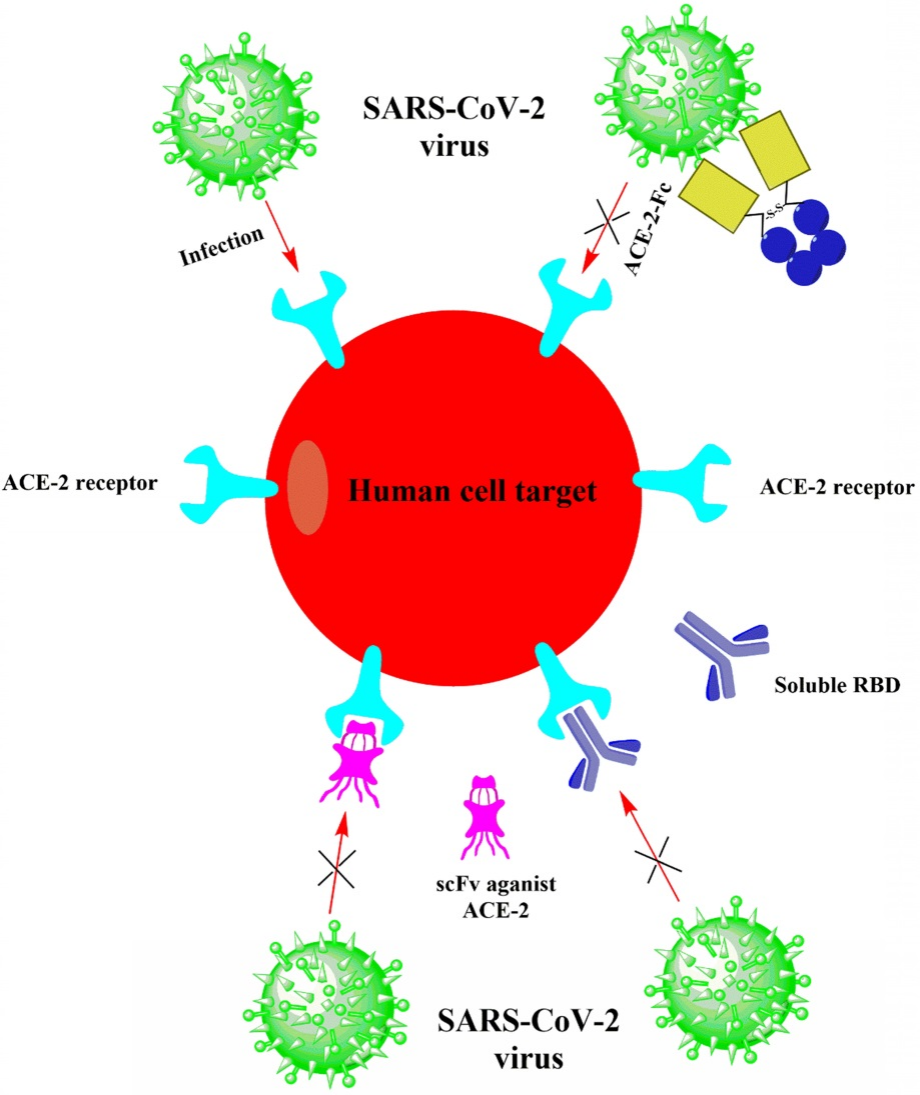
** Suggested therapeutic agents that can be utilized to prevent SARS-CoV-2 from infecting cells.** Target cells expressing ACE2 involve human lung and gastrointestinal tissues. The huge spike protein on the surface of SARS-CoV-2 binds to ACE2 on the infected cell, making the cell to enter. The three suggested procedures would prevent this interaction and thus eliminate the infection. First, the receptor-binding domain (RBD) of the spike protein from SARS-CoV or SARS-CoV-2 will be applied to bind ACE2 and saturate the unoccupied sites. Second, an antibody or a single-chain antibody fragment (scFv) can be applied to ACE2 for the same purpose. The third procedure is to use ACE2 cell outfield directly as bait to bind spike proteins and immediately target coronavirus virions. The Fc domain combined with ACE2 will promote the prolonged cycle of the organism (ACE2-Fc).

Neutralizing antibodies to the spike (S) protein on the surface of SARS-CoV-2 may be the first therapeutic strategy considered by biomedical researchers in academia and industry to provide passive immunity to illness 58. The newly released SARS-CoV-2 genome sequence (GenBank: MN908947.3) enables researchers to conduct gene synthesis in the laboratory and estimate the expression of S proteins as immunogens. Conventional methods of screening mice or rabbits for neutralizing antibodies might be too slow for this outbreak. Still, rapid methods (such as phage or yeast display libraries that express antibody fragments) can be used to identify virally neutralizing antibody candidates quickly 59, 60. The simplest, most direct way to combat SARS-CoV-2 during the outbreak is to interfere with the viral entry into cells, where antibodies usually work 61. It won't be effortless to confirm the functions of neutralizing antibodies quickly. In addition, ensuring that mutated RNA viruses fail to enter the cells will be a challenge. A cocktail of antibodies applied to the Ebola pandemic could be used, but it would add complexity to the production manner 62. However, another strategy that does not rely on directly targeting to viral glycoproteins can apply to the treatment of COVID-19. In this procedure, viral receptor proteins on the host cell surface are neutralized, thereby preventing the virus from binding and entering the cells. Fortunately, investigators have already identified the viral receptors expressed on the cell surface. Up to date, preprint publications have reported that SARS-CoV-2 employs angiotensin- converting enzyme 2 (ACE2) as the receptor for cell entry (Fig. 5) 26, which is the identical receptor that SARS-CoV uses for entry 63. Both CoVs bind to ACE2 through their S proteins on the virions, after which the viral membrane and cell membrane fuse. The RNA virus SARS-CoV-2 then replicates its genome inside the host cell and new virions are eventually produced that are secreted to infect other cells. The fact that both SARS-CoV and SARS-CoV-2 utilize the same ACE2 receptor gives impetus to investigators to develop biologics for SARS-CoV promptly. Based on the SARS reports, some neutralizing antibodies are effective in preventing infections in the SARS prevention models 64.

### SARS-CoV-2 vaccine under development

One important step to control COVID-19 is to develop a vaccine. In the first laboratory stage of a vaccine development, it is necessary to screen the virus strains, attenuate the virus strains as necessary 65. Next steps are to study the adaptability of the virus strains to the cultured cell-matrix and the stability of the virus strains in the passage process, to explore the stability of process quality, and to establish animal models 66. Dr. Paul Stoffels, vice chairman, and chief scientific officer of Johnson & Johnson, is one of the researchers who are engaged in the development of vaccines for SARS-CoV-2. According to his estimate, it takes eight months to a year for the SARS-CoV-2 vaccine to be rigorously tested before it is ready for public use 67. It is also imperative to analyze the rate of SARS-CoV-2 genome evolution and to determine peptide vaccine targets 68. There are eight CD4 T-cell epitopes with high binding affinity within S, E, M, and N proteins that are generally restricted by the human leukocyte antigen (HLA)-DR alleles found in Asian and Asian-pacific populations 69. These immunodominant epitopes can be incorporated into the generic subunit coronavirus vaccine 69. They used highly conserved and annotated structural protein sequences of the representative SARS-CoV-2 strain Wuhan-Hu-1 (MN908947.3) to predict possible CD4 T-cell epitopes 69. Their prediction method recommended by the IEDB is based on the "immune epitope database and analysis resources (IEDB) consensus tool" 69. However, standard mapping epitopes are essential to the design of globally effective subunit vaccines. They, therefore, examined common epitopes that are recognized by all dominant HLA-DR alleles that are prevalent in five ethnic groups 69. Their analyses demonstrated that a subunit vaccine, including eight immunologically dominant HLA-DR epitopes, produced effective antiviral T-cell and antibody responses in populations of different ethnicities 69.

Previous studies have observed that only 23% and 16% of the known SARS-CoV T cell and B cell epitopes respectively are the same as that of SARS-CoV-2 and no mutant sequences have been recognized in the available SARS-CoV-2 epitopes 70. This is a strong evidence of their potential to obtain a potent T cell or antibody response in SARS-CoV-2. In terms of T cells, the identification of the SARS-CoV-derived epitopes, located in the same location as SARS-CoV-2, and the large population assumed to be included, is especially encouraging 70. It proposed further investigation into a vaccine designed to produce a protective T-cell response, which has been proved to afford long-term protection in SARS-CoV 70. Linear SARS-CoV-derived B cell epitopes in the S2 subunit may be more likely applicants for producing protective antibody responses 70. Many of these epitopes, despite their low exposure, map to the same locations as those of SARS-CoV and SARS-CoV-2, and preliminary outcomes have appeared indicating their potential for cross-reactive and antibody neutralization 70. Therefore, vaccine solutions that endeavor to produce antibodies targeting S2 linear epitopes might be useful and should be more investigated.

### Prevention and control

The current research data show that, like SARS-CoV, the SARS-CoV-2 receptor on human host cells is ACE2 26, 71. It is speculated that antibodies or drugs designed for SARS may also be suitable for SARS-CoV-2. Thus they can be used as priority product candidates for disease prevention and treatment. Vaccine research needs to be deployed for a long time. From the experience of SARS-CoV research, the inactivated virus vaccine may have an immunopathological enhancement effect 72. Therefore, adverse reactions should be carefully examined and controlled. One of the candidates is an S-protein receptor-binding region-based vaccine 73. It has a protective effect against SARS-CoV in an experimental animal model. When it comes to small molecular drugs for SARS-CoV-2, several teams from China have carried out the screening of compounds currently on the market as antivirals and found promising results *in vitro*, which are expected to be verified in an experimental animal model and clinical trials 74. Military Academy of Medical Sciences Institute of Chinese Academy of Sciences and Toxic Drug Research Institute jointly found that three drugs, including Remdesivir, Chloroquine, and Ritonavir, inhibited the replication of SARS-CoV-2* in vitro* 75,76. In addition, in 2017, Zhang's group pointed to a host cell protease TMPRSS2 being a potentially important target for the treatment of influenza virus and coronavirus infections in the research on searching and screening the antiviral drugs 77. More recently, results by Hoffmann's group from Germany demonstrate that SARS-CoV-2 spike proteins use the SARS-coronavirus receptor, ACE2, for entry and the cellular protease TMPRSS2 for SARS-CoV-2 spike protein priming 78. A TMPRSS2 inhibitor blocked entry and might constitute a treatment option. Other studies have also shown that yogurt and probiotics can help prevent lung infections in the elderly 79. Since the outbreak of SARS in 2003, China's scientific research capacity in the field of preventive medicine has been dramatically improved. In the outbreak of COVID-19 caused by SARS-CoV-2, rapid progress has been made in etiology identification, diagnostic reagent development, virus characteristic analysis, and clinical treatment strategy. At the same time, popular science education and media publicity have also played a decisive role, and public awareness and social responsibility for the prevention and control of the epidemic have become an essential factor in curbing the rise of the epidemic. However, still much has to be done in this outbreak, which is highlighted by the fact that essential questions, such as the virus's mutation characteristics, transmission pattern, and traceability, have yet to be answered. The scientific and public health community still needs to reflect deeply and act quickly to meet the challenge of SARS-CoV-2 and more new pathogens in the future.

Since ACE2 is a functional receptor of SARS-CoV-2, its safety and potential impact on disease should be fully considered when applying Angiotensin-converting enzyme inhibitors (ACEI) 74. Therefore, for patients with the novel coronavirus pneumonia (COVID-19) combined with hypertension, Calcium channel blockers (CCBs) are recommended if they are taking ACEI or Angiotensin receptor blockers (ARBs) 80, 81. In severe cases, Bradykinin (BK2) receptor blockers or direct renin inhibitor aliskiren can be used 82. If hypotension occurs, antihypertensive drugs should be discontinued 14. The application of ACEI drugs is still controversial and needs further discussion 80. The SARS-CoV-2 causes COVID-19 by infecting host cells via ACE2 receptors, and also causes damage to the myocardium, although the mechanism is unclear. Patients with underlying cardiovascular disease had a poor prognosis after infection with SARS-CoV-2 80, 81. For acute myocardial injury, the primary disease should actively be treated and the myocardial protective treatment carried out 80, 81. For patients with underlying heart disease, especially ST-Elevation Myocardial Infarction (STEMI), early reperfusion therapy should be performed under well-protected conditions. For patients with SARS-CoV-2 infection with cardiovascular disease, they should try to avoid using ACEI or ARB 14, 80, 81.

## Conclusion

The novel coronavirus was first detected in December 2019 during the outbreak of severe pneumonia in Wuhan, Hubei Province, China. The disease caused by the virus outbreak was finally named COVID-19, which stands for the coronavirus (CoV) disease 2019, and the etiologic virus SARS- CoV-2, which stands for severe acute respiratory syndrome CoV 2 by WHO. Scientific investigation is underway to learn more about the virus, its origin, and how it affects humans, and the situation is changing rapidly. Most of the first patients in COVID-19 claimed to be related to the Huanan Seafood market in Wuhan, indicating that the virus had a zoonotic origin. However, the source of the virus remains to be determined. Bats, known as a reservoir for most viruses, might be the original host of the new strain, but the intermediate animal host is unknown. The virus is similar to SARS-CoV-like CoV in bats but is different from SARS-CoV and MERS-CoV. One study suggests that the virus may be a recombination of a bat CoV with a CoV of unknown origin, possibly from snakes 83, but there is no evidence to support this hypothesis. Human-to- human transmission has been confirmed in communities and medical institutions in China, as well as in Vietnam, Japan, Thailand, Germany, and the United States 84, 85. Preliminary assessment of the transmission dynamics of the first 425 confirmed cases found that before January 1, 2020, 55% of the cases were related to the Huanan seafood market in Wuhan; after this date, only 8.6% of the cases were associated with this market 86. This confirms that human-to-human transmission has occurred between close contacts since the middle of December 2019 86. Infections that occurred among health workers were also identified 86. It is unclear how easily the virus can be transmitted from person to person. Asymptomatic carriers can become contagious to others, as there have been confirmed reports of transmission from asymptomatic contacts in Germany 87.

The full genome of the virus has been deposited in GenBank. Molecular assays using Reverse-Transcriptase Polymerase Chain Reaction (RT-PCR) are needed to confirm the diagnosis. There are no specific treatments available for COVID-19 up to now; therefore, the main body of treatment is optimized to support treatment to reduce symptoms and maintain organ functions in more severe diseases. Also, there are no specific anti-SARS-CoV-2 drugs. However, some existing antiviral medications have been used as part of clinical trials 88.

At present, China's National Health Commission has classified the new coronavirus as a class A infectious disease, requiring the most stringent prevention and control measures, including mandatory quarantine of patients and medical observation of those in close contact with them 4. Many Chinese cities are currently on lockdown because of travel bans and the suspension of public transport services. It was reported that most of those who died were the elderly and individuals who had underlying illnesses 89. According to the first case report, the overall case fatality rate is approximately 2.6% 2; however, this estimate should be treated with caution as the actual number of cases of infection and its course are not yet known. We, thus, should carefully observe the current situation and take measures to control COVID-19.

## Figures and Tables

**Table 1:** COVID-19 cases-worldwide. Note: the statistical data as of March 19, 2020.

Continent	Country / Territory / Area	Confirmed cases	Deaths
Africa	Algeria	73	6
Africa	Cameroon	10	0
Africa	Congo	3	0
Africa	Egypt	196	6
Africa	Ethiopia	6	0
Africa	Gabon	3	0
Africa	Ghana	7	0
Africa	Guinea	1	0
Africa	Kenya	7	0
Africa	Mauritania	1	0
Africa	Morocco	54	2
Africa	Namibia	2	0
Africa	Nigeria	8	0
Africa	Rwanda	11	0
Africa	Senegal	36	0
Africa	Seychelles	6	0
Africa	Sudan	2	1
Africa	Togo	1	0
Africa	Tunisia	29	0
Africa	Eswatini	1	0
Africa	Benin	1	0
Africa	Liberia	2	0
Africa	Somalia	1	0
Africa	Burkina Faso	26	1
Africa	Central African Republic	1	0
Africa	Cote dIvoire	6	0
Africa	Democratic Republic of the Congo	14	0
Africa	Equatorial Guinea	3	0
Africa	South Africa	116	0
Africa	United Republic of Tanzania	3	0
Africa	Gambia	1	0
Africa	Djibouti	1	0
Africa	Zambia	2	0
America	Argentina	97	2
America	Bahamas	1	0
America	Bolivia	12	0
America	Brazil	428	4
America	Canada	690	9
America	Chile	238	0
America	Colombia	102	0
America	Cuba	11	1
America	Ecuador	168	3
America	Guatemala	8	1
America	Guyana	5	1
America	Honduras	12	0
America	Jamaica	15	0
America	Mexico	118	0
America	Panama	109	1
America	Paraguay	11	0
America	Peru	145	0
America	Suriname	1	0
America	Uruguay	79	0
America	Venezuela	33	0
America	Antigua and Barbuda	1	0
America	Costa Rica	69	1
America	Dominican Republic	21	0
America	Saint Lucia	2	0
America	Saint Vincent and the Grenadines	1	0
America	Trinidad and Tobago	9	0
America	United States of America	9415	150
America	Barbados	1	0
America	El Salvador	1	0
America	Nicaragua	1	0
Asia	Afghanistan	22	0
Asia	Bahrain	256	1
Asia	Bangladesh	10	0
Asia	Bhutan	1	0
Asia	Cambodia	24	0
Asia	China	81238	3250
Asia	India	165	3
Asia	Indonesia	172	5
Asia	Iran	17361	1135
Asia	Iraq	164	12
Asia	Israel	433	0
Asia	Japan	873	29
Asia	Jordan	52	0
Asia	Kazakhstan	37	0
Asia	Kuwait	142	0
Asia	Lebanon	133	4
Asia	Malaysia	790	2
Asia	Maldives	13	0
Asia	Mongolia	5	0
Asia	Nepal	1	0
Asia	Oman	39	0
Asia	Pakistan	302	0
Asia	Palestine	44	0
Asia	Philippines	202	17
Asia	Qatar	452	0
Asia	Singapore	313	0
Asia	Taiwan	108	1
Asia	Thailand	177	1
Asia	Uzbekistan	23	0
Asia	Vietnam	76	0
Asia	Myanmar	0	0
Asia	Brunei Darussalam	68	0
Asia	Saudi Arabia	171	0
Asia	South Korea	8565	91
Asia	Sri Lanka	42	0
Asia	United Arab Emirates	113	0
Asia	Kyrgyzstan	3	0
Europe	Albania	59	2
Europe	Andorra	53	0
Europe	Armenia	115	0
Europe	Austria	1646	4
Europe	Azerbaijan	34	0
Europe	Belarus	46	0
Europe	Belgium	1486	14
Europe	Bulgaria	92	2
Europe	Croatia	81	0
Europe	Cyprus	58	0
Europe	Denmark	1115	4
Europe	Estonia	258	0
Europe	Finland	369	0
Europe	France	9134	244
Europe	Georgia	34	0
Europe	Germany	8198	13
Europe	Greece	418	5
Europe	Hungary	73	1
Europe	Iceland	250	0
Europe	Ireland	366	2
Europe	Italy	35713	2978
Europe	Kosovo	19	0
Europe	Latvia	71	0
Europe	Liechtenstein	25	0
Europe	Lithuania	33	0
Europe	Luxembourg	210	2
Europe	Malta	48	0
Europe	Moldova	36	1
Europe	Monaco	9	0
Europe	Netherlands	2051	58
Europe	Norway	1423	3
Europe	Poland	287	5
Europe	Portugal	642	2
Europe	Romania	260	0
Europe	Russia	147	0
Europe	Serbia	94	0
Europe	Slovakia	107	0
Europe	Slovenia	286	1
Europe	Spain	13716	598
Europe	Sweden	1301	10
Europe	Switzerland	3010	21
Europe	Turkey	191	1
Europe	Ukraine	19	2
Europe	Bosnia and Herzegovina	36	0
Europe	Czech Republic	522	0
Europe	Holy See	1	0
Europe	North Macedonia	42	0
Europe	San Marino	109	14
Europe	United Kingdom	2630	103
Europe	Montenegro	8	0
Oceania	Australia	565	6
Oceania	New Zealand	28	0
Oceania	French Polynesia	3	0
Oceania	Guam	3	0
Other	Cases on an international conveyance Japan	696	7
Total		213254	8843

**Table 2:** Physicochemical Parameters of SARS-CoV-2.

Parameters	SARS-CoV-2 Sequence ID: (MN908947.3)
Molecular Weight	1069577.59 Da
Number of amino acids	9194
Theoretical pI	9.02
Instability index (II)	43.95 (unstable)
Number of Negatively Charged Residues (Asp + Glu)	560
Number of Positively Charged Residues (Arg + Lys)	971
Aliphatic Index	77.44
Estimated half-life	20 hours (mammalian reticulocytes, *in vitro*) 30 min (yeast, *in vivo*) >10 hours (*Escherichia coli*, *in vivo*)
Grand average of Hydropathicity (GRAVY)	-0.087
